# Does whole-body electrical muscle stimulation combined with strength training promote morphofunctional alterations?

**DOI:** 10.6061/clinics/2019/e1334

**Published:** 2019-10-30

**Authors:** Alexandre Lopes Evangelista, Cauê Vazquez La Scala Teixeira, Bruna Massaroto Barros, Jônatas Bezerra de Azevedo, Marcos Rodolfo Ramos Paunksnis, Cleison Rodrigues de Souza, Tanuj Wadhi, Roberta Luksevicius Rica, Tiago Volpi Braz, Danilo Sales Bocalini

**Affiliations:** I Departamento de Educacao Fisica, Universidade Nove de Julho, Sao Paulo, SP, BR; II Grupo de Estudos da Obesidade, Universidade Federal de Sao Paulo, Sao Paulo, SP, BR; III Programa de Pos Graduacao em Ciencias da Reabilitacao, Universidade Nove de Julho, Sao Paulo, SP, BR; IV Tecfit, Sao Paulo, SP, BR; V Health Sciences and Human Performance, University of Tampa, Tampa, Florida, USA; VI Departamento de Educacao Fisica, Universidade Estacio de Sa, Vitoria, ES, BR; VII Laboratorio de Avaliacao do Movimento Humano, Universidade Metodista de Piracicaba, Piracicaba, SP, BR; VIII Laboratorio de Fisiologia e Bioquimica Experimental, Centro de Educacao Fisica e Esporte, Universidade Federal do Espirito Santo, Vitoria, ES, BR

**Keywords:** Muscle Strength, Resistance Training, Muscle Hypertrophy, Maximal Strength, Neuromuscular Adaptation

## Abstract

**OBJECTIVES:**

The aim of this study was to evaluate the effects of 8 weeks of strength training (ST) combined with whole-body electrical stimulation (EMS) on morphofunctional adaptations in active individuals.

**METHODS:**

Fifty-eight volunteers were randomly distributed into the following groups: an untrained control (UN) group (n=16), an ST group (n=21) or an ST combined with EMS (ST+EMS) group (n=21). Both intervention groups (the ST and ST+EMS groups) performed 3 exercises (biceps curl, back squats and high-pulley tricep extensions) twice a week for 8 weeks. The subjects performed 3 sets of 8 to 12 maximum repetitions (MRs) with a 90-second rest duration between sets. The ST+EMS group performed the resistance training exercises wearing a whole-body suit that provided electrical stimulation at frequencies between 80-85 Hz, with a continuously bipolar impulse duration and pulse breadth of 350 µs. The intensity for each muscle group was controlled by Borg’s category ratio (CR)-10 scale; the intensity started at 5-6 and eventually reached 7-8. One-repetition maximum strength (1RM) and muscle thickness (MT) were measured before and after the training intervention. MT was evaluated in the biceps brachii (BB), triceps brachii (TB), and vastus lateralis (VL).

**RESULTS:**

No differences (*p*>0.05) were found between the ST and ST+EMS groups. Improvements (*p*<0.05) in the absolute values of the morphofunctional parameters after the training protocol were observed. Significant differences were found between both the intervention groups and the UN group (*p*<0.05). The ST+EMS group presented high percentage changes (*p*<0.05) in muscular strength for the 1RM_squat_ (43.2%, ES=1.64) and the MT of the BB (21.6%, ES=1.21) compared to the ST (20.5%, ES=1.43, 11.9%, ES=0.77) group.

**CONCLUSIONS:**

Our data suggest that the combination of ST+EMS may promote alterations in muscle strength and MT in healthy active subjects.

## INTRODUCTION

The regular practice of diverse physical exercise methods is associated with numerous health-related benefits, such as a reduction in body fat (1), the addition of lean mass (2), an improvement in self-esteem (3) and increased functional capacity (4).

As such, varying interventions have been developed to promote motivation to engage in physical exercise (5) and to maximize the results of more traditional interventions. Among these strategies, physical training using whole-body electrical muscle stimulation (EMS) has gained popularity. Whole-body EMS is a relatively new training technology that differs fundamentally from the classic passive and locally applied EMS used for therapeutic (6-9) and sport (10,11) purposes. Modern devices, such as the whole-body suit, have the ability to stimulate all the major muscle groups either in isolation or simultaneously (i.e., up to an area of 2.800 cm^2^) and therefore have been increasingly applied in training programs for health promotion, aesthetic improvement and physical fitness and performance improvement.

The favorable effects of EMS on body composition and physical fitness parameters have been reported in several studies (5). The benefits associated with EMS include the reduction in sarcopenia (12-14), diseases associated with type II diabetes (15) and visceral fat in sedentary individuals with obesity (16). In addition, EMS has been used in a wide variety of populations to improve athletic performance, body composition, functionality and quality of life (1,17).

However, to date, EMS in combination with other interventions to maximize physical training results has not been studied. Among these interventions, strength training (ST) combined with EMS seems to be the most promising since ST is considered the most effective means of increasing strength and muscle tissue hypertrophy (1). Thus, the objective of the present study was to verify the chronic effect of ST plus electrostimulation on the strength and muscle thickness (MT) of physically active subjects. The hypothesis is that the use of EMS during ST will potentiate the effects found with ST alone.

## METHODS

### Experimental Approach to the Problem

A randomized, parallel-group, repeated-measures design was used to investigate the effects of traditional ST, ST combined with whole-body EMS (ST+EMS) and a nontraining control (UN) on morphofunctional adaptations. Both of the training groups (ST and ST+EMS) trained twice a week for 8 weeks. The subjects performed 3 sets of 8 to 12 maximum repetitions with a 90-second rest duration between sets. The total number of sets and repetitions were equal between the groups; however, the ST+EMS group performed the resistance training exercises while wearing a whole-body suit that provided EMS stimuli. Maximum strength and MT were assessed before and after the 8-week training period using one-repetition maximum (1RM) and ultrasonography assessments of the biceps brachii (BB), triceps brachii (TB) and vastus lateralis (VL) muscles.

### Subjects

After approval from the committee of ethics and research of the local institution (protocol number: 2.313.847/2018), sixty-six healthy, physically active subjects volunteered to participate in this study. The exclusion criteria were as follows: subjects with a positive clinical diagnosis of diabetes mellitus, subjects who smoked, and subjects with musculoskeletal complications and/or cardiovascular issues confirmed by medical evaluation.

The volunteers were randomly distributed to one of three groups: an UN group (n=16), an ST group (n=25) or an ST+EMS group (n= 25). During the study period, 8 individuals dropped out of the ST and ST+EMS groups for personal reasons, leaving 21 subjects in each of the two groups included in the statistical analysis, as shown in Table 1. None of the participants had engaged in resistance training for at least six months prior to the experimental period but physically participated in other types of activities (recreational sports and/or endurance training) according to the International Physical Activity Questionnaire (IPAQ).

## METHODS

### Maximum Strength

Maximum dynamic strength was assessed with 1RM testing during biceps curl, back squat and high-pulley tricep extension exercises (Nakagym^®^, São Paulo, Brazil). The testing protocol followed previous recommendations by Haff and Triplett (18). Subjects reported to the laboratory having refrained from any exercise other than activities of daily living for at least 72 hours prior to the testing sessions both before and after the intervention.

In brief, subjects warmed up for 5 minutes on a treadmill (Movement technology^®^, São Paulo, Brazil) at 60% of their maximum heart rate followed by two exercise-specific warm-up sets. During the first set, the subjects performed five repetitions at ∼50% of the estimated 1RM followed by one set of three repetitions at a load corresponding to ∼60-80% of the estimated 1RM, with a 3-minute rest interval between sets. Following the warm-up sets, subjects made five attempts to find their 1RM load, with 3-minute intervals between trials. The 1RM was defined as the maximum weight that could be lifted no more than once with a proper technique. Verbal encouragement was given throughout testing. All the testing sessions were supervised by the research team for validity. The test-retest intraclass correlation coefficients (ICCs) calculated for the data collected during the familiarization and preintervention period for the 1RM_biceps curl_, 1RM_squat_, and 1RM_elbow extension_ were 0.989, 0.990, and 0.988, respectively. The coefficients of variation (CVs) for these measures were 0.8, 0.7, and 0.9%, respectively. The standard errors of measurement (SEMs) for these measures were 2.05, 1.95, and 2.23 kg, respectively.

### Muscle Thickness Assessment

Ultrasonography was used to determine the MT of the biceps brachii and brachialis (BB), TB and VL using an ultrasound-imaging unit (Bodymetrix, BodyMetrix, BX2000, IntelaMetrix, Inc., Livermore, CA) with a wave frequency of 2.5 MHz. The ultrasound probe was applied perpendicular to the skin for measurement. A water-soluble gel was used on the transducer to aid acoustic coupling and remove the need for excess contact pressure on the skin. MT was defined as the distance between the interface of the muscle tissue and subcutaneous fat to the corresponding bone. Imaging was performed on the right side of the body. The subjects were instructed to fast for at least 3 hours prior to testing, and pre- and posttesting assessments were performed at the same time of day.

The BB and TB assessments were performed at a distal point located at 60% of the distance from the lateral epicondyle of the humerus to the acromion process of the scapula. The VL assessments were performed midway between the lateral condyle of the femur and the greater trochanter. For the upper body assessments, the subject’s arms were placed by their sides in a relaxed position while they sat comfortably. For the lower body assessments, the subjects rested in a supine position on an examination bed with their knees fully extended and relaxed. The legs were strapped to each other and to the table to minimize unwanted movement. The MT was assessed by the same blinded researcher pre- and posttest; the researcher was careful to apply minimal pressure when placing the probes on the subject’s skin. To increase test-retest consistency, each site was marked with henna ink and remarked every week. Additionally, in an effort to ensure that swelling of the muscles after training did not obscure the results, images were obtained 48-72 hours before commencement of the study and 48-72 hours after the final training session. This is consistent with research showing that an acute increase in MT returns to baseline within 48 hours of an ST session (19). To further ensure the accuracy of the MT assessments, at least three images were obtained for each region. The test-retest ICCs for the TB, BB, and VL were 0.998, 0.996, and 0.999, respectively. The CVs for these measurements were 0.6, 0.4, and 0.6%, respectively. The SEMs for these measurements were 0.42, 0.29, and 0.41 mm, respectively.

### Familiarization

All the subjects completed two familiarization sessions separated by a minimum of 72 hours before the commencement of the experimental protocol; both sessions occurred one week after the maximum dynamic strength and muscle thickness assessments. During these sessions, subjects were familiarized with the exercises and proper techniques.

### Training Regimens

The subjects underwent a hypertrophy-oriented ST regimen twice a week (at least 48 hours between training sessions) for 8 weeks. The target intensity was 8 to 12 maximum repetitions (MRs) for each exercise. Three sets were performed for each of the following exercises: biceps curl back squats and high-pulley tricep extensions. The exercises were performed with a free repetition tempo, and a 90-second rest interval was allowed between sets. The exercises and repetition schemes remained the same for all 8 weeks in both groups. If a subject was unable to complete the required repetitions, the load was dropped by 2-10% for the upper body and 2-15% for the lower body exercises. On the other hand, if a subject was able to perform one or two more repetitions (i.e., 13-14 repetitions), the load was increased by 2-10% for the upper body and 2-15% for lower body exercises (20). Each training session lasted approximately 20 minutes.

The ST+EMS group performed the same training regimen with the addition of whole-body EMS provided by a suit (XBody^®^, Dorsten, Nordrhein-Westfalen, Germany). The EMS suit stimulated 5 muscle groups during the ST exercises (the biceps during the biceps curl exercise; the quadriceps, hamstrings and glutes during the back squat exercises; and the triceps during the high-pulley tricep extension exercises). The intensity of the EMS current progressively increased during the interventional period (Table 2). The subjects were asked to rate the average intensity of the EMS session and the regional intensity of the EMS on a rating scale (Ratings of Perceived Exertion [RPE]); the intensity was maintained between 5-6 for weeks 1 and 2 and increased to 7-8 for weeks 3 to 8.

The subjects were asked to refrain from performing any type of additional exercise regimen throughout the study duration. Research staff supervised all training sessions, provided verbal encouragement and ensured that the subjects performed the correct number of sets and repetitions with the correct exercise technique.

### Statistical Analyses

The normality and homogeneity of the data were verified using the Shapiro-Wilk and Levene tests, respectively. Prior to analysis, all the data were log-transformed to reduce bias arising from nonuniformity errors (heteroscedasticity). The means, SDs and 95% confidence intervals (CIs) were calculated for the normally distributed data. A repeated-measures 2x3 analysis of variance (ANOVA) was used to compare the 1RM_biceps curl_, 1RM_squat_, 1RM_elbow extension_, and MT of the BB, TB and VL using the groups as fixed factors and the subjects as random factors. Post hoc comparisons were performed with a Bonferroni correction. Assumptions of sphericity were evaluated using Mauchly’s test; if sphericity was violated (*p*<0.05), the Greenhouse-Geisser correction factor was applied. In addition, effect sizes (ESs) were evaluated using a partial eta squared (η^2^_p_), with <0.06, 0.06 to 0.14 and >0.14 indicating a small, medium, and large effect, respectively. Cohen’s d was calculated as the difference in the means divided by the pooled standard deviation (
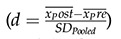
) to measure the absolute differences (pre *vs* posttest) in the raw values of the variables (4). The results from Cohen’s d were qualitatively interpreted using the following thresholds: <0.2, trivial; 0.2 - 0.6, small; 0.6 -1.2, moderate; 1.2 - 2.0, large; 2.0 - 4.0, very large and; >4.0, extremely large. If the 90% confidence limits overlapped, small positive and negative values for the magnitude were deemed unclear; otherwise, the observed magnitude was deemed acceptable (10). The trivial area (d<0.2, gray bar) is marked in forest plot graph. All analyses were conducted using SPSS-22.0 (IBM Corp., Armonk, NY, USA). The adopted significance was *p*≤0.05.

## RESULTS

As shown in Table 3, significant main effects of time (*F_1,15_*=74.437, *p*<0.001, η^2^ *_p_*=0.832) and the group x time interaction (*F_2,30_*=26.666, *p*<0.001, η^2^ *_p_*=0.640) were observed for the 1RM_biceps curl_. There were significant differences in time (*F_1,15_*=214.970, *p*<0.001, η^2^ *_p_*=0.935) and the group x time interaction (*F_2,30_*=59.405, *p*<0.001, η^2^ *_p_*=0.798) for the 1RM_squat_. Significant main effects of time (*F_1,15_*=72.417, *p*<0.001, η^2^ *_p_*=0.828) and the group x time interaction (*F_2,30_*=24.021, *p*<0.001, η^2^ *_p_*=0.616) were observed for the 1RM_elbow extension_.

Significant main effects of time (*F_1,15_*=46.977, *p*<0.001, η^2^ *_p_*=0.758) and the group x time interaction (*F_2,30_*=27.510, *p*=0.038, η^2^ *_p_*=0.521) were observed for the BB. There were significant differences in time (*F_1,15_*=115.319, *p*<0.001, η^2^ *_p_*=0.885) and the group x time interaction (*F_2,30_*=34.458, *p*=0.038, η^2^ *_p_*=0.703) for the TB. Significant main effects of time (*F_1,15_*=73.302, *p*<0.001, η^2^ *_p_*=0.830) and the group x time interaction (*F_1.343,20.142_*=11.196, *p*=0.038, η^2^ *_p_*=0.427) were observed for the VL, as shown in Table 4.

**Figure 1:**
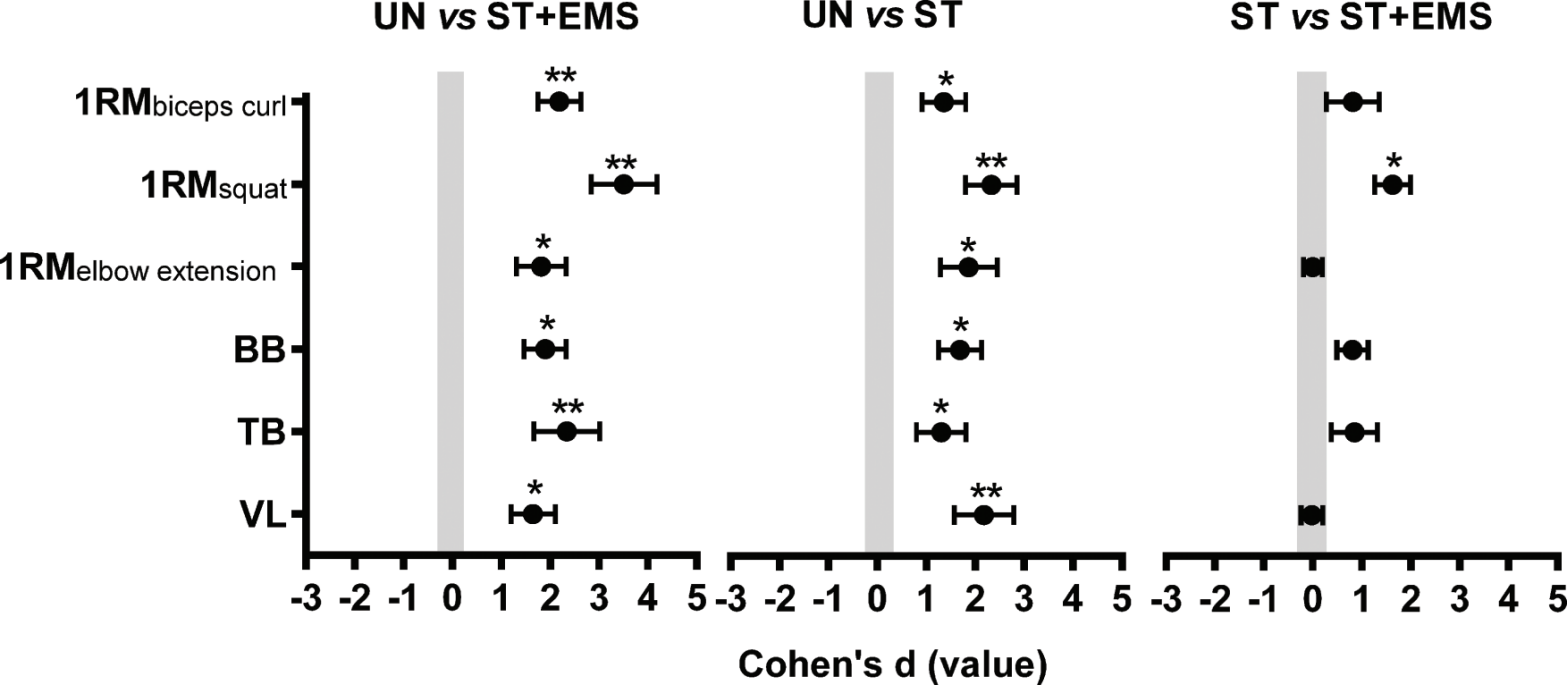
Coheńs effect size (ES) principle ± 90% confidence intervals (CIs) were used to compare the absolute differences between the untrained (UN), strength training (ST) and strength training combined with body electric stimulation (ST+EMS) groups considering the one-repetition maximal strength test (1RM) and the muscle thickness (MT) of the biceps brachii and brachialis (BB), triceps brachii (TB) and vastus lateralis (VL). *Large ES **Very large ES.

Figure 1 represents the results of the Cohen’s d ESs. The absolute differences after 8 weeks of training between the UN *vs* ST+EMS group were large for the 1RM_elbow extension_ (d=1.82, 90% CI=1.31 to 2.33), BB (d=1.90, 90% CI=1.47 to 2.33) and VL (d=1.65, 90% CI=1.20 to 2.10) and very large for the 1RM_biceps curl_ (d=2.19, 90% CI=1.75 to 2.63), 1RM_squat_ (d=3.51, 90% CI=2.84 to 4.18) and TB (d=2.34, 90% CI=1.06 to 3.02).

The differences between the UN *vs* ST groups were large for the 1RM_biceps curl_ (d=1.36, 90% CI=0.91 to 1.81), 1RM_elbow extension_ (d=1.87, 90% CI=1.24 to 2.45), BB (d=1.69, 90% CI=1.25 to 2.13) and TB (d=1.31, 90% CI=0.80 to 1.82) and very large for the 1RM_squat_ (d=2.33, 90% CI=1.80 to 2.86) and VL (d=2.18, 90% CI=1.57 to 2.79). In comparison, the ESs of the ST *vs* ST+EMS groups were moderate for the 1RM_biceps curl_, BB and TB (d<1.2), favoring the ST+EMS group. A large ES for the 1RM_squat_ was found in the ST *vs* ST+EMS groups (d=1.63, 90% CI=1.27 to 1.99).

## DISCUSSION

The present study aimed to investigate the chronic effects of 8 weeks of ST combined with EMS on maximal strength and MT in physically active individuals. To the best of our knowledge, this is the first study to analyze traditional ST in combination with EMS. EMS has been applied with different approaches, such as in therapeutic (6-9), sports (10-11) practices, and has shown positive results in reducing sarcopenia (15), diabetes (12) and obesity in sedentary individuals with obesity (16).

The results showed that both training protocols (ST and ST+EMS) resulted in a significant improvement in all the studied variables (strength and MT) compared to the control group (*p*<0.05 for all variables). However, although ANOVA revealed no significant differences between the training protocols, percentage changes and effect sizes in the ST+EMS group presented higher gains for elbow flexor strength when compared to the ST group (Δ%=24.3%, ES=0.77 *vs*. Δ%=15.1%, ES=0.50, respectively). The results also suggest that the strength of the lower limbs can benefit from the addition of EMS (ST+EMS: Δ%=43.2%, ES=1.64 *vs*. ST: Δ%=20.5%, ES=1.43).

Regarding muscle hypertrophy, there were greater percentage gains and effect sizes for the BB and TB variables in the ST+EMS group than in the ST group (ST+EMS BB: Δ%=21.6%, ES=1.21 *vs* ST BB: Δ%=1.9%, ES=0.77, and ST+EMS TB: Δ%=16.8%, ES=0.97 *vs* ST TB: Δ%=9.1%, ES=0.64), similar to strength.

These findings are in line with the study by Ahmad and Hasbullah (21), who demonstrated gains in both strength and muscle mass by subjecting 15 physically active individuals to 5 weeks of EMS training. The sessions lasted for 20 minutes twice a week, similar to those in this study. According to the authors, the addition of EMS to resistance training generated an increase in the mechanical stress and, consequently, in the pattern of recruitment of the motor units.

Increased mechanical stress has been postulated as one of the main stimuli for the process of myofibrillar protein synthesis and consequent muscle hypertrophy (22). In addition, the possible additional recruitment of muscle fibers resulting from the combination of EMS and ST can maximize energy expenditure and metabolic stress. In this case, metabolic stress has been noted as one of the factors that contributes to the increase in the cross-sectional area of the muscle (23).

Kemmler et al. (23) pointed out that the addition of EMS to resistance training seemed to be effective in relation to muscular adaptation. The authors also compared the effects of traditional training (a combination of resistance exercises plus endurance exercises performed 2 times a week) with training combined with EMS (traditional training plus 20 minutes of EMS). The results of the study demonstrated that the 20-minute exercise plus EMS regimen was more effective for strength gains and lean mass maintenance than traditional isolation training. Thus, the authors considered the application of this new exercise technology as an alternative for individuals interested in increasing the functional and morphological adaptations obtained from ST. These data show that EMS is an effective means of physical training that focuses on neuromuscular and morphological adaptations and should be considered as an option by fitness instructors and those interested in rehabilitation.

In the present study, it should be noted that although the ST+EMS group presented increased percentage changes and ESs for muscle strength and thickness, there was no significant difference between the interventions, leading us to believe that a longer intervention time is necessary for significant differences to be observed in the study population. This hypothesis needs to be tested in future studies. Other limitations are also important to consider. We had a relatively small sample size, and no EMS control group was included. Additionally, caloric intake is an important factor; although all the subjects in the study were instructed not to alter their dietary intake and/or caloric consumption during the training intervention, these data were not collected or strictly monitored. Another important point to note is that the subjects were physically active but did not regularly participate in resistance training; therefore, these results should be considered with caution when applying them to individuals who engage in resistance training.

Despite these limitations, our data suggested positive alterations in the morphofunctional parameters and warrant further research on effective EMS practices and the effects of different training method combinations in various populations. However, some limitations should be addressed; the sample size was relatively small, and this was a short-term study with no information about long-term outcomes. Despite the significant induction of positive changes found in this study, more studies are needed to examine the effects of EMS in different healthy populations.

Our findings showed that the combination of EMS and ST did not harm muscular adaptations after 8 weeks. As such, strength and conditioning professionals as well as fitness instructors working with healthy untrained subjects may consider adding EMS to regular ST regimens as a strategy for muscular adaptation.

## AUTHOR CONTRIBUTIONS

Each author made significant individual contributions to this manuscript. Evangelista AL and Teixeira CVLS conceived the study, acquired and interpreted the data, and drafted the manuscript. Barros BM, de Azevedo JB and Wadhi T interpreted the data and drafted and reviewed the manuscript. Souza CR conceived the study, acquired and interpreted the data. Paunksnis MRR, Rica RL and Braz TV analyzed the data and reviewed the manuscript. Evangelista AL and Bocalini DS conceived the study, acquired and interpreted the data, drafted and reviewed the manuscript.

## Figures and Tables

**Table 1:** Sample characteristics.

Parameters	UN	ST	ST+EMS
Age (years)	27.1±4.1	25.1±3.2	25.5±6.1
Body mass (kg)	78.8±12.9	78.1±15.3	78.1±7.5
Height (cm)	177±0.08	175±0.07	176±0.06

Values are expressed as the means ± standard deviations (SDs) for the untrained control (UN), strength training (ST) and strength training combined with EMS (ST+EMS) groups.

**Table 2:** Electrical stimulation protocol.

Program variables	Weeks 1 and 2	Weeks 3 to 8
Stimulation frequency	80 Hz	85 Hz
Impulse duration	continuously	continuously
Pulse breadth	350 µs	350 µs
Impulse type	bipolar	bipolar
Duration	∼20 minutes	∼20 minutes
Regional intensity (Borg’s CR-10 scale)	5-6	7-8

**Table 3:** Muscle strength parameters after 8 weeks of training.

Parameters	Pre	Post	Δ%	Cohen’s d	ANOVA 3x2	
					time	time*group
				ES	*p* value	*p* value
1RM*_biceps curl_* (kg)						
UN	33±9	33±9	2.7	0.10	0.176	
ST	33±10	38±10^a^ #	15.1	0.50	<0.001	
ST+EMS	34±10	43±12^a^ #	24.3	0.77	<0.001	<0.001
1RM*_squat_* (kg)						
UN	92±11	91±11	-1.1	-0.09	0.292	
ST	92±12	111±14^a^ #	20.5	1.43	<0.001	
ST+EMS	93±18	133±30^a^ #	43.2	1.64	<0.001	<0.001
1RM*_elbow extension_* (kg)						
UN	27±5	27±6	1.6	0.08	0.269	
ST	27±9	33±10^a^ #	22.1	0.62	<0.001	
ST+EMS	28±7	34±6^a^ #	21.2	0.90	<0.001	<0.001

Values are expressed as the mean ± the standard deviation (SD) for the untrained (UN), strength training (ST) and strength training combined with whole-body EMS (ST+EMS) groups. One-repetition maximal strength (1RM). Effect size (ES).

a Significant (*p*<0.05) differences compared to before training.

# Significant (*p*<0.05) differences compared to the UN group.

**Table 4:** Muscle thickness (MT) measurements after 8 weeks of training.

Parameters	Pre	Post	Δ%	Cohen’s d	ANOVA 3x2	
					time	time*group
				ES	*p* value	*p* value
BB (mm)						
UN	34.0±3.3	34.4±3.4	1.2	0.12	0.214	
ST	32.6±7.1	36.5±4.8^a^ #	11.9	0.77	<0.001	
ST+EMS	33.2±6.1	40.4±5.8^a^ #	21.6	1.21	<0.001	0.038
TB (mm)						
UN	33.5±3.0	34.0±3.1	1.4	0.16	0.248	
ST	33.1±4.3	36.1±5.1^a^ #	9.1	0.64	<0.001	
ST+EMS	31.5±5.3	36.8±5.6^a^ #	16.8	0.97	<0.001	0.038
VL (mm)						
UN	40.9±5.2	41.0±5.4	0.4	0.03	0.142	
ST	41.4±5.5	46.8±4.6^a^ #	13.0	1.06	<0.001	
ST+EMS	40.9±5.9	46.2±5.2^a^ #	12.9	0.95	<0.001	0.038

Values expressed as the mean ± standard deviation (SD) for the untrained (UN), strength training (ST) and strength training combined with body electric stimulation (ST+EMS) groups. Muscle thickness (MT) of the biceps brachii and brachialis (BB), triceps brachii (TB) and vastus lateralis (VL). Effect size (ES).

a Significant (*p*<0.05) differences compared to before.

# Significant (*p*<0.05) differences compared to the UN group.
